# Testing the effect of high-definition transcranial direct current stimulation of the insular cortex to modulate decision-making and executive control

**DOI:** 10.3389/fnbeh.2023.1234837

**Published:** 2023-09-28

**Authors:** Irene Gorrino, Nicola Canessa, Giulia Mattavelli

**Affiliations:** ^1^IUSS Cognitive Neuroscience (ICoN) Center, Scuola Universitaria Superiore IUSS, Pavia, Italy; ^2^Istituti Clinici Scientifici Maugeri IRCCS, Cognitive Neuroscience Laboratory of Pavia Institute, Pavia, Italy

**Keywords:** high-definition tDCS, insula, executive control, decision-making, loss/risk aversion, treatment

## Abstract

**Introduction:**

Previous neuroimaging evidence highlighted the role of the insular and dorsal anterior cingulate cortex (dACC) in conflict monitoring and decision-making, thus supporting the translational implications of targeting these regions in neuro-stimulation treatments for clinical purposes. Recent advancements of targeting and modeling procedures for high-definition tDCS (HD-tDCS) provided methodological support for the stimulation of otherwise challenging targets, and a previous study confirmed that cathodal HD-tDCS of the dACC modulates executive control and decision-making metrics in healthy individuals. On the other hand, evidence on the effect of stimulating the insula is still needed.

**Methods:**

We used a modeling/targeting procedure to investigate the effect of stimulating the posterior insula on Flanker and gambling tasks assessing, respectively, executive control and both loss and risk aversion in decision-making. HD-tDCS was applied through 6 small electrodes delivering anodal, cathodal or sham stimulation for 20 min in a within-subject offline design with three separate sessions.

**Results:**

Bayesian statistical analyses on Flanker conflict effect, as well as loss and risk aversion, provided moderate evidence for the null model (i.e., absence of HD-tDCS modulation).

**Discussion:**

These findings suggest that further research on the effect of HD-tDCS on different regions is required to define reliable targets for clinical applications. While modeling and targeting procedures for neuromodulation in clinical research could lead to innovative protocols for stand-alone treatment, or possibly in combination with cognitive training, assessing the effectiveness of insula stimulation might require sensitive metrics other than those investigated here.

## Introduction

Previous neuroimaging evidence has consistently shown the role of the insular cortex in conflict monitoring and decision-making (Canessa et al., 2013, 2017; Markett et al., 2016). Several studies have reported the involvement of this region in a wide array of functions, placing it at the interface between interoception, emotion and cognition (Chang et al., 2013; Uddin et al., 2017; Molnar-Szakacs and Uddin, 2022). In particular, insular activity related to interoceptive representation, performance monitoring, as well as awareness of emotional states and body movement, has been interpreted in terms of bodily physiological signals guiding decision-making based on the outcome of behavioral learning processes (Craig, 2009). This hypothesis was supported by studies investigating the neural bases of decision-making under risk and/or uncertainty, showing increased activation of the right insular cortex when making riskier compared to safer choices (Paulus et al., 2003) and higher anticipatory insular response preceding riskless choices (Kuhnen and Knutson, 2005).

Based on anatomical, functional and connectivity features, the insula has been parceled in multiple subregions, and particularly in posterior and anterior sections divided by the central insular sulcus (Wysiadecki et al., 2018). The anterior section, highly connected with frontal and limbic areas, has been mainly related to emotional and cognitive functions, whereas the posterior insula (PI) plays a crucial role in interoceptive processing and behavioral regulation via connections to posterior-temporal and parietal areas, as well as sensorimotor cortices (Cloutman et al., 2012; Centanni et al., 2021). These sectors appear to underpin a posterior-to-anterior flow of neural signals, whereby the progressive integration of sensory information into mechanisms of “salience” detection underpins the modulation of cognitive control and goal-directed behavior by sensory-interoceptive and affective stimuli (Craig, 2009; Centanni et al., 2021). Accordingly, neuroimaging studies have shown the PI role in representing homeostatic states related to the experience of risk, in turn shaping upcoming decisions (Xue et al., 2010). One such experience concerns the mental anticipation and evaluation of choices entailing both positive and negative prospective outcomes, typically associated with the overweighting of potential losses compared with gains, and thus with the individual’s tendency to prefer avoiding losses to acquiring equivalent gains (i.e., loss aversion; Kahneman and Tversky, 1979). Previous neuroimaging evidence shows that individual differences in the extent of loss aversion reflect the strength of activation of multiple brain structures, including the striatal and dorsal anterior cingulate (dACC) sectors of the meso-cortico-limbic pathway (Tom et al., 2007), and the right PI—extending into the supramarginal gyrus—both when making real choices (Canessa et al., 2013) and at rest (Canessa et al., 2017).

Consistent with the role of the insula in loss aversion, clinical evidence shows that operculo-insular resection for drug-resistant epileptic seizures impairs patients’ sensitivity to expected value when facing a potential loss during decision-making under risk (Von Siebenthal et al., 2017). Moreover, insular damage has been associated to a reduction of the typical gambling-related cognitive distortions in tasks simulating real-life gambling (Clark et al., 2014): compared both to controls and patients with lesions in the amygdala or ventromedial prefrontal cortex (vmPFC), those with lesions confined to insula did not show the so-called near-miss effects (i.e., higher motivation to play a gamble after non-wins close to the jackpot) or gambler’s fallacy (i.e., the belief that non-recent outcomes are more likely to happen).

By showing a role of the insula in modulating adaptive behavioral learning via sensory-affective signals, these data suggest its possible involvement in conditions characterized by poor adaptation to changing environments. This view is supported by growing evidence of structural and functional abnormalities, in this region, in patients with major depressive disorder (Herwig et al., 2010; Surguladze et al., 2010; Takahashi et al., 2010; Stratmann et al., 2014), bipolar disorder (Ellison-Wright and Bullmore, 2010; Hulvershorn et al., 2012; Wise et al., 2017), anxiety disorders (Stein et al., 2007; Terasawa et al., 2013), schizophrenia (Wylie and Tregellas, 2010), psychopathy for abnormal socio-emotional processing (Blair, 2013), anorexia nervosa (Kaye et al., 2009), addiction (Ibrahim et al., 2019), as well as different deficits associated with neurological disorders (Löffler et al., 2016; Namkung et al., 2017). Overall, this evidence highlights the importance of assessing insular responsiveness to tasks tapping evaluation and control as a potential translational biomarkers (or “endophenotypes”; Sharp et al., 2012) of adaptive behavioral learning, that might also represent the target of innovative neuromodulation protocols with therapeutical purposes (Downar et al., 2016).

Notably, several studies indeed showed strong functional connectivity between the PI and the dACC (Taylor et al., 2009; Ghaziri et al., 2017), i.e., a key node of the “executive” network (Shenhav et al., 2016), which highlights a possible neural basis of the insula role in cognitive control via salience processing (Dosenbach et al., 2007; Seeley et al., 2007). This notion highlights the potential translational implications of investigating the role of insula and dACC, since both regions map strikingly well with a common core of brain areas involved in different psychiatric conditions (Goodkind et al., 2015; Downar et al., 2016), that might be targeted in innovative treatment protocols. Since this aim can be pursued with non-invasive neurostimulation and neuromodulation techniques, studies investigating their feasibility are required to pave the way to possible clinical applications (Downar et al., 2016). Transcranial direct current stimulation (tDCS) is a relatively inexpensive, portable and well tolerated candidate for this purpose (Brunoni et al., 2012), especially with high-definition montages (HD-tDCS), that are expected to improve the intensity and/or focality of stimulation compared with the “conventional” bipolar approach (Edwards et al., 2013; Huang et al., 2019). It is worth noting that optimization methods for HD-tDCS highlighted a non-trivial trade-off between focality and variability of the effects with different montages (Dmochowski et al., 2011; Mikkonen et al., 2020; Lee et al., 2021). The available evidence from computational models suggests that 4 × 1 ring montages (i.e., one small stimulating electrode surrounded by a ring of four small reference electrodes) grant higher focality, but at the cost of larger inter-individual variability in strength and distribution of electric fields (Mikkonen et al., 2020; Van Hoornweder et al., 2022). On the other hand, model-based HD-tDCS montages produce larger current dispersion, which is however compensated by higher stimulation intensity at the target site (Lee et al., 2021), resulting in an overall optimal trade-off between focality, individual variability, and intensity (Mikkonen et al., 2020).

By using a model-based approach, we previously reported evidence of the significant effect of dACC stimulation, via HD-tDCS, on cognitive control and loss aversion in decision-making under risk (Mattavelli et al., 2022). It is still unknown, instead, whether such modulation might be also elicited by HD-tDCS of the insula. Since this area is a potential key target for the treatment of different neuropsychiatric conditions (Downar et al., 2016), this is a critical gap that should be filled to investigate its feasibility and its effects.

On this basis, we assessed the effects of anodal and cathodal HD-tDCS over the right PI in modulating executive control on the Flanker task, and both loss and risk aversion in well-established tasks of decision-making under risk (Kahneman and Tversky, 1979). We replicated the experimental paradigms and methodological procedures used in our previous study (Mattavelli et al., 2022), in which decreased “Flanker” conflict effect and increased loss/risk aversion suggested an improvement of conflict monitoring—involving both visual-attentional skills and behavioral control—after cathodal HD-tDCS of the dACC. As mentioned above, the dACC and PI are highly connected, and the activity of both regions has been reported to track individual differences in response inhibition (Wager et al., 2005; Fedota et al., 2016) and loss aversion (Canessa et al., 2013). We therefore predicted that active HD-tDCS of the right PI, compared to sham stimulation, might confirm the modulation of behavioral performance in the Flanker and gambling tasks observed in our previous study.

## Materials and methods

### Participants

Twenty-five healthy right-handed participants (15 females) aged 20–26 years [mean 22.6; standard deviation (SD): 1.87] took part in the study. Three participants were excluded due to inconsistencies in their performance, leading to a total of 22 participants. In detail, the model for estimating loss aversion did not converge for one participant, while two participants showed loss aversion lambda (λ) > 10, suggestive of the tendency to reject all gambles (see the method below). We replicated the experimental design, power analysis and sample size used in our previous study (Mattavelli et al., 2022). Namely, we planned to perform a repeated measures ANOVA on the Flanker conflict effect, by entering in G*Power (Faul et al., 2007) the effect size computed from a study with a similar design (Weiss and Lavidor, 2012; partial η2 = 0.17). This procedure resulted in an estimated sample size of 19 participants assessed in three separate sessions (α = 0.05, 1 − β = 0.9). Notably, the observed effect size from our first experiment was in line with, and even larger than, this previous evidence (partial η2 = 0.197), indicating that a sample of 22 participants should enable finding modulatory effects.

Participants’ eligibility was assessed with a questionnaire considering the following exclusion criteria for brain stimulation studies (Keel et al., 2001): (1) diagnosis of, or familiarity with, epilepsy; (2) susceptibility to, or history of, migraine; (3) history of neurological or psychiatric disorders; (4) history of brain surgery, tumor or intracranial metal implantation (such as hearing aids, pacemakers or metal plates near the face); (5) current use of psychiatric drugs or psychoactive medications; (6) presence of pacemaker or other implanted devices; (7) diagnosis of specific learning disabilities (e.g., dyslexia); (8) current pregnancy. Participants provided their written informed consent to the experimental procedure, previously approved by ICS Maugeri Ethics Committee, that was based on the latest version of the declaration of Helsinki and on tDCS safety guidelines (Poreisz et al., 2007).

### Experimental procedure

The experiment was based on a single-blind cross-over design. Participants received three stimulation sessions with either active anodal, active cathodal or sham HD-tDCS, separated by a wash-out period of at least 3 days. Although findings regarding the greater effectiveness of offline vs. online stimulation are still controversial (Martin et al., 2014), the former appears to result in greater modulation of task performance (Friehs and Frings, 2019). Based on this evidence, and in keeping with our previous study (Mattavelli et al., 2022), we used an offline approach to assess the neurostimulation effect of HD-tDCS on the right PI. The order of both stimulation conditions (anodal, cathodal, sham) and experimental tasks (Flanker, Loss aversion, Risk aversion) was randomized to control for potential effects of task order or participants’ fatigue.

During the training phase, participants were informed that their performance on the three tasks would led to increase or decrease an initial monetary endowment. Both stimulation and tasks were then performed in a shielded silent cabin, where participants were seated on a comfortable chair and were asked to remain still and relaxed in front of a high-frequency (148 Hz) LCD screen, placed at a 65 cm viewing distance. Each stimulation session lasted 20 min, during which participants were presented a series of short videoclips previously rated as emotionally neutral (Samson et al., 2016). Since the duration of tDCS aftereffects is known to at least equate the duration of stimulation (Batsikadze et al., 2013; Renzi et al., 2015; To et al., 2018; Mattavelli et al., 2019, 2022), the three tasks (Flanker, Loss Aversion, and Risk Aversion) were started immediately after stimulation, so as to be completed within 20 min. The experimental tasks were presented via Presentation software,^1^ while responses were recorded throughout a keyboard. At the end of each session, participants were asked to fill a questionnaire addressing potential HD-tDCS cutaneous or adverse effects (Fertonani et al., 2015). Finally, after the last session, they were also asked to report which sessions they believed were real or placebo, and to express the degree of confidence of their judgment on a scale from 1 to 10 (1 = not certain at all; 10 = completely certain).

### High-definition transcranial direct current stimulation

**Figure 1:**
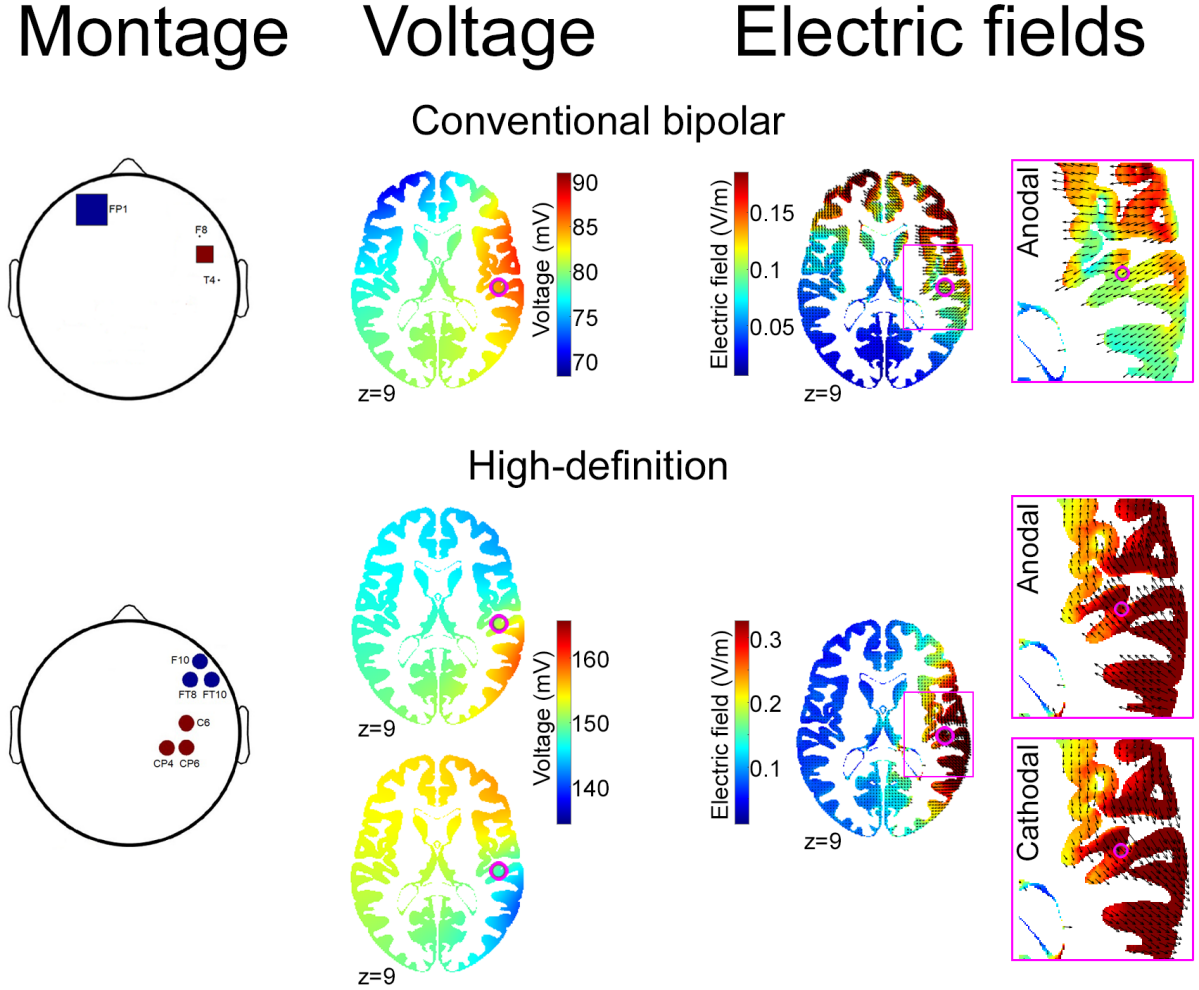
Conventional bipolar, and optimized HD, tDCS montages. The figure depicts tDCS montages aimed to stimulate the right posterior insular cortex using a previously reported conventional bipolar approach (Sagliano et al., 2019; top) and an optimized electrode configuration generated by the ROAST toolbox (Huang et al., 2016; bottom) to provide maximum stimulation intensity in the stereotactic coordinates that have been previously associated with neural loss aversion [xyz: 48 −21 9 (pink dot); Canessa et al., 2013]. For each configuration, the figure depicts the montage [number, shape and size of anode (red) and cathode (blue) electrodes; left], alongside the resulting voltage (middle) and electric field (right) distribution. For HD-tDCS, the right-most panels show the opposite direction of current flows—maximally involving the right posterior insula—for anodal and cathodal montages.

The ROAST toolbox (Huang et al., 2016) was used to identify the optimal montage for maximum intensity of stimulation over the insula. Specifically, the montage was set to target the right PI coordinates where activity was previously reported to track individual difference in loss aversion (Canessa et al., 2013, 2017). The montage was optimized by modeling 6 mm radius circular electrodes (DaSilva et al., 2015) together with the indexes of conductivity assigned to different tissues based on a high resolution T1-weighted MRI image (Huang et al., 2016). The resulting optimal solution consisted of 3 anodes and 3 cathodes, placed on CP4-C6-CP6 and FT8-F10-FT10, respectively. This montage was then modeled to check for the current flow location and intensity when placing 9.5 mm radius electrodes and conductive gel (Figure 1). Each anode and cathode electrode acts as a source and sink, for a total delivered current of 3 mA (i.e., 1 mA current intensity each; current density of 0.35 mA/cm2). The electrodes were placed into saline-soaked sponges of the same shape and size, and placed on the participants’ scalp with the help of an EEG brain cap that was used to correctly identify the electrode locations based on the 10–10 system. The HD montage was implemented through 3 triggered battery-driven neurostimulation devices (Brainstim, EMS Italy).

During anodal sessions, stimulation was performed through a constant current of 1 mA for each anode electrode, that was maintained for 20 min, gradually ramping up in intensity for the first 15 s and slowly decreasing down to 0 mA for the last 15 s. The same holds for the cathodal stimulation, but with reversed polarity. These parameters are in line with safety guidelines for transcranial electric stimulation (tDCS) in humans (Nitsche et al., 2003a, 2004; Gandiga et al., 2006). For the sham sessions, intensity gradually ramped up at the beginning of the stimulation, was maintained for the first 30 s, and then slowly decreased to 0 mA; it was then increased again for the final 30 s of the stimulation. This protocol was adopted to ensure participants’ blinding to the stimulation type (Gandiga et al., 2006; Poreisz et al., 2007; Palm et al., 2013), without inducing an after-effect (Nitsche et al., 2003b; Woods et al., 2016).

### Tasks and stimuli

#### Flanker task

**Figure 2:**
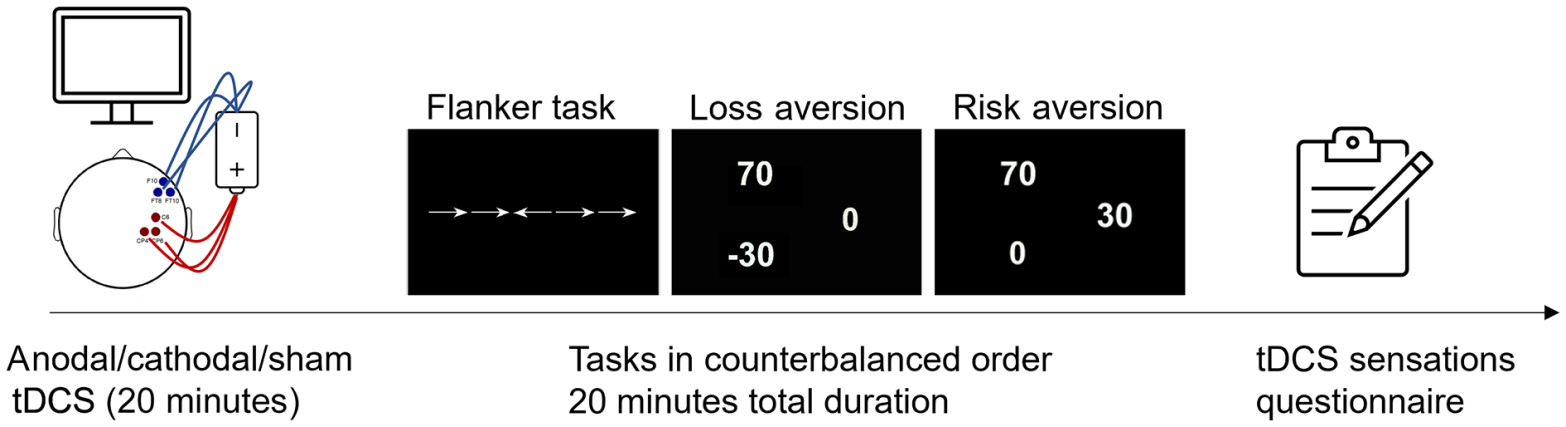
The figure depicts one representative tDCS session, with examples of stimuli from the three experimental tasks (presented in counterbalanced order across participants), i.e., from left to right: an incongruent trial of the Flanker task, and gambling tasks requiring to choose between (a) a certain outcome and a risky mixed-gamble resulting in two equally probable (*p* = 50%) gain-loss outcomes (loss aversion task), or (b) a gamble with equally probable variable positive or 0 outcomes and a certain but smaller gain (risk aversion task).

Participants were asked to indicate the left or right direction of a central horizontal arrow by pressing the corresponding key on a keyboard as quickly and accurately as possible. Three conditions were presented (32 trials each, for a total of 96 trials) in which arrows could either appear surrounded by (1) horizontal lines with no symbolic value—neutral condition; (2) horizontal arrows with the same direction as the target arrow—congruent condition; (3) horizontal arrows with opposite direction to the target—incongruent condition (Figure 2). To assess executive control, Fan et al.’s (2002) “conflict effect” measure was adopted, by subtracting the mean RT of congruent conditions from the mean RT of incongruent condition. The resulting difference is a measure of participants’ susceptibility to the conflict effect, typically reflecting in slower RTs when responding to incongruent than congruent flankers (Fan et al., 2002). Arrows could either appear above or below the fixation point, with the target arrow being preceded by several possible visual cues: no cue, spatial cue, central cue, double cue. While spatial cues could indicate the position of the target arrow (above or below the fixation point), central and double cues were ambiguous. While this manipulation would enable to calculate alerting and orienting effects (Fan et al., 2002), the limited number of trials—due to time constraints related to the duration of the stimulation—only allowed to focus on the conflict effect. Each trial included a fixation point (400 ms), followed or not by the visual cue (100 ms), another fixation point (400 ms), and then the target stimuli (1,700 ms). Trials were presented in random order and were separated by a blank screen of variable duration (either 1,450, 1,500, or 1,550 ms). The eye-monitor distance and the size of the stimuli were computed to obtain a visual angle of 0.588 for each arrow, and 0.068 for the distance between successive arrows (Fan et al., 2002, 2005; Mattavelli et al., 2022).

#### Loss and risk aversion tasks

To assess individual differences in loss and risk aversion, and their possible modulation by HD-tDCS, participants were asked to perform two gambling tasks that required evaluating real prospective monetary gains and losses. In each trial of both tasks, participants had to choose between a risky gamble resulting in one of two equally probable (*p* = 50%) outcomes, and a certain alternative. The Loss Aversion task entailed 50 gain-loss trials during which participants were asked to choose between (a) a certain 0 outcome (i.e., the status quo) and (b) a gamble that might result—with equal probabilities—in either a gain or a loss. The Risk Aversion task included 50 gain-only trials requiring to choose between (a) a gamble with equal probabilities of leading to a gain or 0, and (b) a certain but smaller gain (Figure 2). In both tasks, gambles were presented for 4,500 ms, with the inter-trial-interval ranging between 1,450 and 1,550 ms (Mattavelli et al., 2022). Unbeknownst to participants, the gain-loss ratio in the Loss Aversion tasks, and the gain-certain outcome ratio in the Risk Aversion task, were trial-wise adjusted based on participants’ previous responses. Such adjustment was based on a staircase algorithm (Fleming and Dolan, 2010; Takahashi et al., 2012; Takeuchi et al., 2016; Chen et al., 2020; Mattavelli et al., 2022), through which the initial ratio, set at 2.5, either increased or decreased when the participant rejected or accepted a gamble, respectively. This adjustment had a step-size of 0.5/n, where “n” is the number of trials after the last reversal between accepting or rejecting the gamble. Stimuli were sampled from a symmetric gain-loss matrix ranging between 1 and 100, in steps of 1 (Mattavelli et al., 2022). This procedure was aimed at progressively reducing the range of the subjective indifference point, i.e., the gain-loss (or risky-certain outcome) ratio corresponding to the maximum decisional conflict, associated with a 50% probability of accepting/rejecting the gamble (Mattavelli et al., 2022). To avoid possible learning effects and focus on “decision utility” (i.e., “pure” anticipation without the expectation of outcome delivery; Canessa et al., 2013, 2017; Tom et al., 2007), gambles were not solved immediately. Instead, participants were informed that one among the accepted gambles would be randomly extracted and played by the computer, leading to increase or decrease the monetary reward in case of win or loss, respectively.

The individual degree of loss and risk aversion were estimated, for each subject and stimulation session, by simultaneously fitting both gain-loss and gain-only trials to the following Prospect-theory-inspired model (Sokol-Hessner et al., 2015):


$$\mathrm{Pr}\left(\mathrm{accept}\;\mathrm{gamble}|G,\;L,\;B\right)=\frac{1}{1+e^{-\mu \times \left(p_{G}\times {\left(G\right)}^{\rho }-\lambda \times p_{L}\times {\left(-L\right)}^{\rho }-B^{\rho }\right)}}$$

where G is the gain (G > 0), L is the loss (L < 0 for gain-loss gambles and L = 0 for gain-only gambles), B the guaranteed gain (B = 0 for gain-loss gambles and B > 0 for gain-only gambles), pG = 0.5 is the probability of a gain and pL = 1 − pG = 0.5 is the probability of a loss. The free parameters of the model are: (a) the loss aversion lambda (λ), i.e., the multiplicative weight associated with anticipated losses compared with gains; (b) the risk attitude rho (ρ), i.e., the curvature of the value function u(x) = x^ρ that embodies the diminishing sensitivity to increasing outcome; and (c) the choice consistency or “softmax temperature” (μ), i.e., a measure of noisiness vs. systematicity in choices. Since λ is positively skewed, its natural logarithm—ln(λ)—was modeled in the statistical analyses. Exclusion criteria were (a) a lack of convergence after 50,000 iterations, indicating the lack of model fitting and thus representing inconsistent choices; (b) λ >10, suggestive of the tendency to reject all gambles (Arioli et al., 2023).

## Results

### tDCS tolerability and blinding

Responses to the questionnaire on tDCS-related sensations were analyzed as in our previous study (Mattavelli et al., 2022). Supporting the effectiveness of the blinding procedure, there was no difference across anodal, cathodal or sham sessions concerning (a) the proportion of participants reporting that they received a real stimulation [86%, 86%, and 73%, respectively; χ^2^(2) = 1.83, *p* = 0.4], and (b) the confidence in this judgment [*F*(2, 42) = 2.89, *p* = 0.07]. Subjective sensations were generally low. Only the burning sensation was significantly different across condition, with higher ratings in the cathodal compared to the sham session (*post hoc* Bonferroni corrected *p* = 0.04), while all the other sensations were not rated differently across sessions (see Supplementary Table S1).

### Flanker task

**Figure 3:**
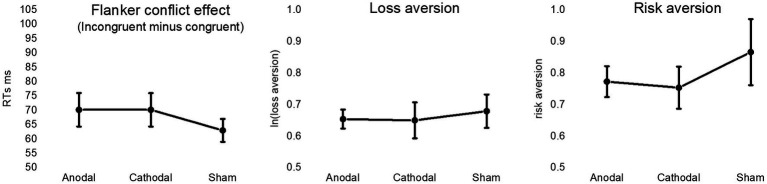
The figure depicts mean values in anodal, cathodal and sham HD-tDCS stimulation session for the conflict effect associated with the differential response time to incongruent and congruent trials of the Flanker task (left panel), the natural logarithm of the degree of loss aversion (central panel), and the degree of risk aversion (right panel). Vertical bars represent standard error of the means.

The conflict effect was computed as the differential RTs to incongruent and congruent trials (Fan et al., 2005), after excluding for each participants the trials with RTs greater than 2 standard deviations above the individual mean (Figure 3). To assess the evidence for, or against, the presence of HD-tDCS modulatory effects, a Bayesian statistical approach was adopted using JASP software (Version 0.16; Westfall et al., 1997; Rouder et al., 2012; Morey and Rouder, 2015). Analyses with frequentist statistics were also performed, and reported in Supplementary material. In line with the classification scheme from the JASP guidelines (van Doorn et al., 2021), Bayes Factor (BF) between 1 and 3 is considered weak/anecdotal evidence, BF between 3 and 10 is considered moderate evidence, and BF > 10 is considered strong evidence, in favor of the alternative hypothesis. On the other side, BF between 1 and 1/3 indicates weak/anecdotal evidence, BF between 1/3 and 1/10 indicates moderate evidence, and BF < 1/10 indicates strong evidence, in support of the null hypothesis.

A Bayesian repeated-measures analysis of variance (ANOVA) with default prior (r scale fixed effects = 0.5; r scale random effects = 1) was used to test the effect of HD-tDCS on executive control, by introducing conflict effect as dependent variable and stimulation condition (anodal, cathodal and sham) as independent variable.

The Mean conflict effect was 70.17 ms (SD = 27.46), 70.17 ms (SD = 27.34), and 62.99 ms (SD = 18.77) in the anodal, cathodal and sham sessions, respectively. Bayesian ANOVA provided moderate support to the null hypothesis (BF_10_ = 0.28), thus indicating no differential modulation of Flanker performance by stimulation conditions.

For the sake of completeness, we also performed analyses on both (a) RTs to different trial types (congruent, incongruent, neutral), and (b) the effect of the previous trial on congruent/incongruent trials (congruency sequence effects; see Egner, 2007). Their results (reported in Supplementary material) confirmed the absence of HD-tDCS modulation on these outcome variables.

### Loss and risk aversion

The mean degree of loss aversion was 1.94 (SD = 0.28), 1.99 (SD = 0.67) and 2.05 (SD = 0.80) following anodal, cathodal and sham stimulation, respectively. Mean risk aversion was 0.77 (SD = 23), 0.75 (SD = 0.31) and 0.86 (SD = 0.49) in the anodal, cathodal and sham sessions, respectively. For both indexes, Bayesian repeated-measures ANOVA with default prior provided moderate support to the null hypothesis, i.e., BF_10_ = 0.15 for loss aversion and BF_10_ = 0.21 for risk aversion (Figure 3). The analysis on choice consistency (i.e., the slope of the logistic regression curve) provided anecdotal evidence for the null hypothesis (BF_10_ = 0.4), being the mean index of uncertainty 1.10 (SD = 1.20), 1.23 (SD = 1.62), and 1.54 (SD = 1.62) in the anodal, cathodal condition and sham sessions, respectively. Results from frequentist statistical analyses are reported in Supplementary material.

## Discussion

We aimed to assess the effect of HD-tDCS of the right PI on different facets of cognitive control such as the conflict effect on the Flanker task, and both loss and risk aversion in a decision-making task based on mixed gambles, in healthy individuals.

To the best of our knowledge, this is the first study using a modeling/targeting approach to modulate insular activity with HD-tDCS, that allows to maximize stimulation intensity at the target site, within safety parameters and with good tolerability (Huang et al., 2019). To this purpose, we replicated the experimental procedure adopted in a previous study that proved successful in modulating performance in the same tasks following HD-tDCS of the dACC (Mattavelli et al., 2022). There are both scientific and translational implications of investigating the feasibility and the effects of modulating PI activity: (i) strong connectivity between insula and dACC (Medford and Critchley, 2010; Ghaziri et al., 2017); (ii) previous evidence of an asymmetric anticipatory response of activation for losses and deactivation for gains reflecting behavioral loss aversion (i.e., “neural loss aversion”; Tom et al., 2007; Canessa et al., 2013, 2017); (iii) the translational relevance of targeting the insula, which, alongside the dACC, has been highlighted as a common neural substrate for several neuropsychiatric conditions (Downar et al., 2016).

While cathodal stimulation of the dACC increased behavioral control both in the Flanker and gambling tasks (Mattavelli et al., 2022), no such effect was found, in the present study, when targeting the right PI. Bayesian statistics rather provided moderate evidence in favor of the null model for the Flanker conflict effect and for both loss and risk aversion, along with anecdotal evidence for the null model concerning the “choice consistency” index. Overall, these results are suggestive of no significant modulation of performance in these tasks by HD-tDCS of the right PI.

As mentioned in the Introduction, the modeling/targeting procedure that we applied is expected to maximize the intensity of the stimulation on the target (Huang et al., 2016) and to decrease the individual heterogeneity of tDCS effects (Mikkonen et al., 2020; Lee et al., 2021), although the electric field modeling showed some current dispersion in temporo-parietal regions (Figure 1). This represents a limitation of the model-based HD-tDCS approach, since, in absence of direct measures of the physiological effect of stimulation, we cannot rule out the interpretation that results might reflect a suboptimal modulation of the insula, or counterproductive behavioral effects of electric fields affecting the cortical regions around the target. Our previous findings on the significant effect of dACC stimulation (Mattavelli et al., 2022) however support the effectiveness of targeting regions located in the depth of sulci, and thus suggest that the null present findings might rather indicate that the right PI—despite its strong connectivity with dACC (Ghaziri et al., 2017)—is not an effective target for modulating cognitive control or decision-making. However, this claim needs further investigation to be confirmed, using neurophysiological methods or combined neurostimulation and neuroimaging techniques (e.g., Meinzer et al., 2012; Pisoni et al., 2018; Esmaeilpour et al., 2020; Vergallito et al., 2023), which would allow disentangling the hypotheses that such null findings reflect the lack of impact on these tasks despite an effective insular modulation, or rather the ineffective modulation of the cortical target.

In the case of the Flanker conflict effect, another potential target is the anterior insula, where previous neuroimaging studies identified, both in normal (Trautwein et al., 2016) and pathological (Galandra et al., 2018, 2019, 2021; Crespi et al., 2019; Canessa et al., 2021, 2022) conditions, subregions involved in attentional control and salience processing that might act as a gatekeeper in executive control (see Molnar-Szakacs and Uddin, 2022). Based also on the high connectivity between posterior and anterior insula (Craig, 2009), the latter might be targeted by further studies to investigate a modulation of performance in tasks tapping executive control, including the Flanker conflict effect.

The lack of a significant modulation of participants’ loss and risk aversion is instead more unexpected, based both on our previous evidence of “neural loss aversion” in the right PI (Canessa et al., 2013, 2017), and on other neuroimaging (Xue et al., 2010; Purcell et al., 2021) and lesional (Clark et al., 2014) data showing its involvement in risky decision making. One possible interpretation for this null result concerns the use of a single-session stimulation, that has been questioned by meta-analytic evidence, particularly for cognitive tasks (Horvath et al., 2015; Medina and Cason, 2017). Single sessions are indeed more vulnerable to the unwanted impact of individual differences in susceptibility to stimulation and brain plasticity (Berryhill and Martin, 2018; Vergallito et al., 2022). Since multiple-sessions studies are more robust in this respect (Elmasry et al., 2015; Berryhill, 2017; see Berryhill and Martin, 2018), future studies should investigate whether repeated sessions of HD-tDCS stimulation over the insula allow overcoming individual variability in responsiveness to induce significant modulatory effects. This is not only relevant, but also more common than single-session protocols, in clinical contexts, which might benefit from usual multi-session protocols to improve symptoms in conditions associated with altered insular activity (Downar et al., 2016; Ibrahim et al., 2019). Other types of electric stimulation protocols, such as transcranial alternating current stimulation (tACS) and transcranial random noise stimulation (tRNS), have been reported as effective in modulating cortical activity, but, to the best of our knowledge, there are no studies applying these protocols to target the insula. In particular, theta-tACS seems promising in modulating executive functions (Klink et al., 2020), while there is limited evidence on the modulation of higher cognitive functions by tRNS, that has been mainly tested on motor and perceptual tasks (van der Groen et al., 2022; Brancucci et al., 2023). Thus, both the outcomes of tACS and tRNS on executive control and decision-making, and their effectiveness in targeting the insula, represent open research lines.

On the other hand, the importance of addressing the technical feasibility of insula stimulation is supported by recent multifaceted evidence of its effect on interoceptive accuracy (Sagliano et al., 2019) and compassion motivation when attending to another’s pain (Di Bello et al., 2023). Both these studies used a bipolar montage with electrodes placed over the frontotemporal region, that—as shown by computational modeling (see Figure 1)—induced larger electric fields over the frontal cortex compared to our high-density montage. This finding highlights the non-trivial trade-off between the effects of maximizing the intensity of stimulation via the targeting procedure vs. spreading electric fields in wide portions of the cortex at the cost of further decreased focality when bipolar montages are applied. Other studies targeted the insula with alternative neurostimulation techniques, such as transcranial magnetic stimulation (TMS) with H-coils, that, while generally associated with some discomfort (Levkovitz et al., 2009; Kaster et al., 2018), is considered a safe procedure allowing to reach deep brain regions (Zangen et al., 2005; Roth et al., 2007). Repetitive TMS with H-coil over the insula has been reported to reduce dopamine levels in substantia nigra and striatum (Malik et al., 2018), and to affect functional connectivity with the medial prefrontal cortex (Lee et al., 2020) and the default mode network (Moeller et al., 2022). Another study targeting the insula with H-coils in a sample of patients with anorexia nervosa reported a significant reduction of symptoms related to obsessions and compulsions, as well as depression and anxiety scores (Knyahnytska et al., 2019). In contrast, a study applying a single session in healthy volunteers reported the absence of significant modulations on blink-suppression and risk behavior (Spagnolo et al., 2019). Overall, these findings highlight inconsistent data on the effects of insula stimulation, which should therefore be interpreted with caution because of methodological variability across studies.

In conclusion, we assessed for the first time the modulatory effect of HD-tDCS applied on the right PI based on a modeling/targeting procedure. Apart from the target region, we replicated a methodological procedure that proved effective in decreasing the Flanker conflict effect and increasing both loss and risk aversion after cathodal stimulation of the dACC (Mattavelli et al., 2022). Bayesian statistical analyses provided moderate support for the absence of modulatory effects on executive control and decision-making variables following anodal or cathodal stimulation of the right PI. While confirming the technical feasibility of stimulating this region with a HD-tDCS approach grounded in targeting and modeling procedures, this negative finding suggests that further research is needed to unveil the factors shaping its actual effects and the tasks that are best suited to measure them, to increase the efficacy of translational applications.

## Data availability statement

The raw data supporting the conclusions of this article will be made available by the authors, without undue reservation.

## Ethics statement

The studies involving humans were approved by Ethics Committee of ICS Maugeri—Pavia Institute. The studies were conducted in accordance with the local legislation and institutional requirements. The participants provided their written informed consent to participate in this study.

## Author contributions

GM and NC conceptualized and designed the study. IG and GM collected the data, performed the statistical analysis, and wrote the first draft of the manuscript. IG organized the database. NC wrote sections of the manuscript. All authors contributed to the article and approved the submitted version.

## Funding

This research was partially supported by the “Ricerca Corrente” funding scheme of the Italian Ministry of Health.

## Conflict of interest

The authors declare that the research was conducted in the absence of any commercial or financial relationships that could be construed as a potential conflict of interest.

## Publisher’s note

All claims expressed in this article are solely those of the authors and do not necessarily represent those of their affiliated organizations, or those of the publisher, the editors and the reviewers. Any product that may be evaluated in this article, or claim that may be made by its manufacturer, is not guaranteed or endorsed by the publisher.

## Footnotes

^1^ www.neurobs.com
