# Supplementary material for: Testing the effect of high-definition transcranial direct current stimulation of the insular cortex to modulate decision-making and executive control
Source: Front Behav Neurosci. 2023 Sep 28;17:1234837. doi: 10.3389/fnbeh.2023.1234837 (PMC10568024; doi:10.3389/fnbeh.2023.1234837)
Supplement: Supplementary file 1 [file Data_Sheet_1.docx]

**Testing the effect of high-definition transcranial direct current stimulation of the insular cortex to modulate decision-making and executive control**

Irene Gorrino^1^, Nicola Canessa^1,2^, Giulia Mattavelli^1,2^

*1 IUSS Cognitive Neuroscience (ICoN) center, Scuola Universitaria Superiore IUSS, Pavia, 27100, Italy*

*2 Istituti Clinici Scientifici Maugeri IRCCS, Cognitive Neuroscience Laboratory of Pavia Institute, 27100, Italy*

**Supplementary Table**

Table S1. Mean values (and standard deviation) of different tDCS-sensations resulting from a questionnaire filled after each session. The second line in each cell shows the percentage of participants reporting a value > 2 (i.e., considerable or strong sensation).

| Session | Itchiness | Pain | Burning | Heat | Pinching | Iron taste | Fatigue |
| --- | --- | --- | --- | --- | --- | --- | --- |
| Anodal | 1.82 (0.96)  22% | 0.5 (0.74)  0% | 0.95 (0.84)  4% | 0.41 (0.79)  4% | 1.27 (0.98)  13% | 0.09 (0.23)  0% | 0.5 (0.67)  0% |
| Cathodal | 1.95 (1)  27% | 0.5 (0.74)  0% | 1.27 (0.93)  9% | 0.32 (0.78)  4% | 1.64 (1)  22% | 0.05 (0.21)  5% | 0.5 (0.8)  0% |
| Sham | 1.45 (0.96)  9% | 0.45 (0.67)  0% | 0.73 (0.88)  4% | 0.32 (0.48)  0% | 1.23 (0.75)  0% | 0 (0)  0% | 0.59 (0.73)  0% |
| Across session statistics | F(2,42)=2.03, p=.14 | F(2,42)=0.09, p=.91 | F(2,42)=3.35, p=.045 | F(2,42)=0.15, p=.86 | F(2,42)=2.71, p=.08 | F(2,42)=1.54, p=.23 | F(1.34,28.27)=0.24, p=.7 |

**Supplementary Results**

**Frequentist analysis**

A repeated-measures analysis of variance (ANOVA) on Flanker “conflict effect” showed non-significant main effect of HD-tDCS on RT [F(2, 42)=1.08, p=.35, partial eta-squared (η2p)=0.046, 95% CI=0-0.19].

The ANOVAs testing the effect of HD-tDCS on decision-making tasks showed non-significant effect for LA [F(1.188, 24.953)=0.28, p=.64, η2p=0.013, 95% CI=0-0.18] and RA [F(1.398, 29.352)=0.56, p=.52, η2p=0.026, 95% CI=0-0.19] indexes.

**Flanker RTs for trial type**

A Bayesian repeated-measures ANOVA on RTs with factors tDCS (anodal, cathodal, sham) and trial type (congruent, incongruent, neutral) showed that the data were more likely to occur under the model including only the main effect of trial rather than models including the effect of tDCS, and the inclusion BF for each specific effect provided strong support for not including tDCS main effect and tDCS-by-trial effect (See Table S2 and S3 for BF on model comparison and analysis of effect). As expected, RTs were slower for incongruent than congruent and neutral trials (Figure S1).

Consistently, a frequentist repeated-measures ANOVA highlighted the significant main effect of trial type [F(2, 42)= 250.03, p<.001, η2p=0.923, 95% CI=0.87-0.94], while the tDCS main effect [F(2, 42)= 0.29, p=.74, η2p=0.014, 95% CI=0-0.11] and tDCS-by-trial type interaction [F(2.708, 56.867)= 0.91, p=.43, η2p=0.041, 95% CI=0-0.14] were not significant.

Figure S1. RTs for trial type. Error bars represent 95% credible intervals.


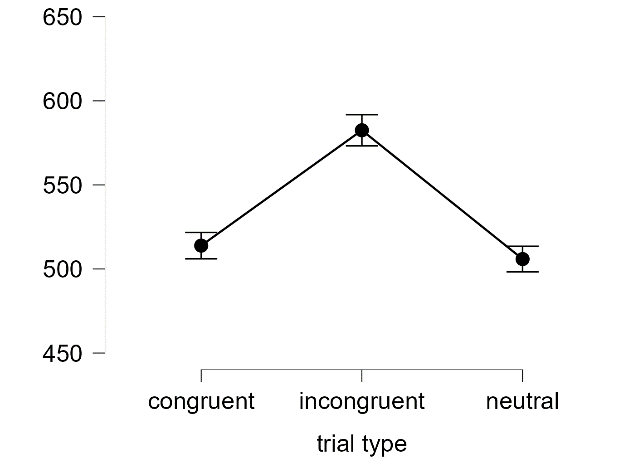


Table S2. Model comparison (to the best model) for tDCS by trial type ANOVA.

| **Models** | | **P(M)** | | **P(M\|data)** | | **BF_M_** | | **BF_10_** | | **error %** | |
| --- | --- | --- | --- | --- | --- | --- | --- | --- | --- | --- | --- |
| trial |  | 0.200 |  | 0.896 |  | 34.361 |  | 1.000 |  |  |  |
| tdcs + trial |  | 0.200 |  | 0.100 |  | 0.443 |  | 0.111 |  | 1.584 |  |
| tdcs + trial + tdcs ✻  trial |  | 0.200 |  | 0.005 |  | 0.019 |  | 0.005 |  | 2.765 |  |
| Null model (incl. subject) |  | 0.200 |  | 7.897e-34 |  | 3.159e-33 |  | 8.816e-34 |  | 1.088 |  |
| tdcs |  | 0.200 |  | 5.610e-35 |  | 2.244e-34 |  | 6.263e-35 |  | 1.352 |  |

Table S3. Analysis of effects for tDCS by trial type ANOVA.

| **Effects** | | **P(incl)** | | **P(excl)** | | **P(incl\|data)** | | **P(excl\|data)** | | **BF_incl_** | |
| --- | --- | --- | --- | --- | --- | --- | --- | --- | --- | --- | --- |
| tdcs |  | 0.600 |  | 0.400 |  | 0.104 |  | 0.896 |  | 0.078 |  |
| trial |  | 0.600 |  | 0.400 |  | 1.000 |  | 5.884e-15 |  | 1.133e+14 |  |
| tdcs ✻  trial |  | 0.200 |  | 0.800 |  | 0.005 |  | 0.995 |  | 0.019 |  |

**Flanker “congruency sequence” effect**

The congruency sequence effect was analysed considering RTs for congruent and incongruent trials, further divided on the basis of the previous trial (i.e. congruent after congruent, congruent after incongruent, incongruent after congruent, incongruent after incongruent). These RTs were introduced in a Bayesian repeated-measures ANOVA with factors tDCS (anodal, cathodal, sham), trial type (congruent, incongruent), and previous trial (congruent, incongruent). The analysis showed that the data were more likely to occur under the model including only the main effect of trial rather than models including the effect of tDCS, and the inclusion BF for each specific effect provided strong support for not including tDCS as main effect or in interaction with other factors. Notably, there is inconclusive evidence for the inclusion of trial type-by-previous trial interaction (BF_inclusion_=0.68), suggesting the possible role of the congruency sequence effect (See Table S4 and S5 for BF on model comparison and analysis of effect). Indeed, RTs were slower for incongruent than congruent trials, with RTs for congruent trials further decreased when preceded by congruent trials (Figure S2).

This observation was confirmed by the same analysis performed with frequentist approach, which showed the significant main effect of trial type [F(1, 21)= 215.65, p<.001, η2p=0.911, 95% CI=0.81-0.94] and significant trial type-by-previous trial interaction [F(1, 21)= 21.33, p<.001, η2p=0.504, 95% CI=0.17-0.68], while all the other main effects and interactions were not significant (all ps>.35).

Figure S2. RTs for trial type divided by previous trial condition. Error bars represent 95% credible intervals.


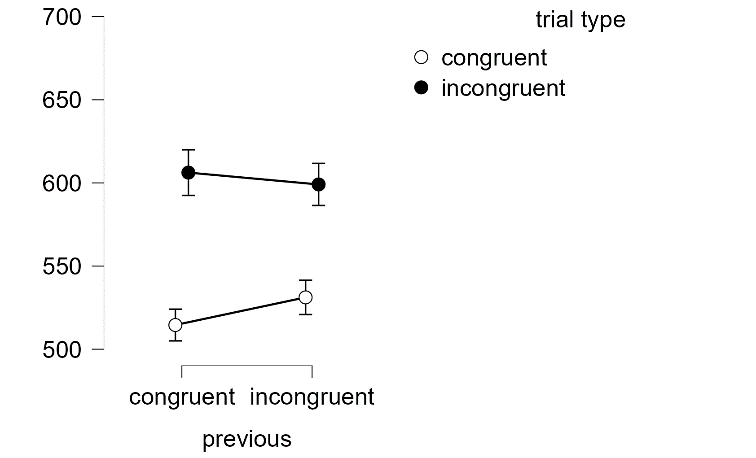


Table S4. Model comparison (to the best model) for tDCS by trial type by previous trial ANOVA.

| **Models** | | **P(M)** | **P(M\|data)** | | **BF_M_** | | | **BF_10_** | | **error %** | |
| --- | --- | --- | --- | --- | --- | --- | --- | --- | --- | --- | --- |
| trial type |  | 0.053 |  | 0.451 |  | 14.787 |  | 1.000 |  |  |  |
| tdcs + trial type |  | 0.053 |  | 0.166 |  | 3.573 |  | 0.367 |  | 2.155 |  |
| trial type + previous + trial type ✻  previous |  | 0.053 |  | 0.164 |  | 3.529 |  | 0.363 |  | 8.139 |  |
| trial type + previous |  | 0.053 |  | 0.088 |  | 1.746 |  | 0.196 |  | 2.497 |  |
| tdcs + trial type + previous + trial type ✻  previous |  | 0.053 |  | 0.063 |  | 1.203 |  | 0.139 |  | 4.008 |  |
| tdcs + trial type + previous |  | 0.053 |  | 0.034 |  | 0.626 |  | 0.075 |  | 3.821 |  |
| tdcs + trial type + tdcs ✻  trial type |  | 0.053 |  | 0.015 |  | 0.270 |  | 0.033 |  | 2.215 |  |
| tdcs + trial type + previous + tdcs ✻  previous + trial type ✻  previous |  | 0.053 |  | 0.007 |  | 0.125 |  | 0.015 |  | 3.685 |  |
| tdcs + trial type + previous + tdcs ✻  trial type + trial type ✻  previous |  | 0.053 |  | 0.005 |  | 0.094 |  | 0.012 |  | 2.452 |  |
| tdcs + trial type + previous + tdcs ✻  previous |  | 0.053 |  | 0.004 |  | 0.065 |  | 0.008 |  | 2.737 |  |
| tdcs + trial type + previous + tdcs ✻  trial type |  | 0.053 |  | 0.003 |  | 0.054 |  | 0.007 |  | 2.940 |  |
| tdcs + trial type + previous + tdcs ✻  trial type + tdcs ✻  previous + trial type ✻  previous |  | 0.053 |  | 7.816e-4 |  | 0.014 |  | 0.002 |  | 11.147 |  |
| tdcs + trial type + previous + tdcs ✻  trial type + tdcs ✻  previous |  | 0.053 |  | 3.417e-4 |  | 0.006 |  | 7.576e-4 |  | 3.297 |  |
| tdcs + trial type + previous + tdcs ✻  trial type + tdcs ✻  previous + trial type ✻  previous + tdcs ✻  trial type ✻  previous |  | 0.053 |  | 1.083e-4 |  | 0.002 |  | 2.400e-4 |  | 3.704 |  |
| Null model (incl. subject) |  | 0.053 |  | 7.396e-34 |  | 1.331e-32 |  | 1.640e-33 |  | 1.561 |  |
| previous |  | 0.053 |  | 1.178e-34 |  | 2.120e-33 |  | 2.612e-34 |  | 1.752 |  |
| tdcs |  | 0.053 |  | 9.678e-35 |  | 1.742e-33 |  | 2.146e-34 |  | 1.863 |  |
| tdcs + previous |  | 0.053 |  | 1.518e-35 |  | 2.733e-34 |  | 3.367e-35 |  | 2.086 |  |
| tdcs + previous + tdcs ✻  previous |  | 0.053 |  | 1.456e-36 |  | 2.620e-35 |  | 3.228e-36 |  | 2.812 |  |

Table S5. Analysis of effects for tDCS by trial type by previous trial ANOVA.

| **Effects** | | **P(incl)** | | **P(excl)** | | **P(incl\|data)** | | **P(excl\|data)** | | **BF_incl_** | |
| --- | --- | --- | --- | --- | --- | --- | --- | --- | --- | --- | --- |
| tdcs |  | 0.737 |  | 0.263 |  | 0.297 |  | 0.703 |  | 0.151 |  |
| trial type |  | 0.737 |  | 0.263 |  | 1.000 |  | 0.000 |  | ∞ |  |
| previous |  | 0.737 |  | 0.263 |  | 0.369 |  | 0.631 |  | 0.208 |  |
| tdcs ✻  trial type |  | 0.316 |  | 0.684 |  | 0.024 |  | 0.976 |  | 0.054 |  |
| tdcs ✻  previous |  | 0.316 |  | 0.684 |  | 0.012 |  | 0.988 |  | 0.026 |  |
| trial type ✻  previous |  | 0.316 |  | 0.684 |  | 0.240 |  | 0.760 |  | 0.683 |  |
| tdcs ✻  trial type ✻  previous |  | 0.053 |  | 0.947 |  | 1.083e-4 |  | 1.000 |  | 0.002 |  |
